# The Prevalence of Myopia in Children in Spain: An Updated Study in 2020

**DOI:** 10.3390/ijerph182312375

**Published:** 2021-11-25

**Authors:** Cristina Alvarez-Peregrina, Clara Martinez-Perez, Cesar Villa-Collar, Mariano González-Pérez, Ana González-Abad, Miguel Ángel Sánchez-Tena, on behalf of Grupo de Investigación Alain Afflelou

**Affiliations:** 1 Faculty of Biomedical and Health Science, Universidad Europea de Madrid, 28670 Madrid, Spain; cristina.alvarez@universidadeuropea.es (C.A.-P.); villacollarc@gmail.com (C.V.-C.); 2 ISEC Lisboa, Instituto de Educação e Ciência de Lisboa, 1750-179 Lisboa, Portugal; masancheztena@ucm.es; 3 Department of Optometry and Vision, Faculty of Optics and Optometry, Universidad Complutense de Madrid, 28037 Madrid, Spain; mgonzalezperez@afflelou.es; 4 Training and Development Department, Alain Afflelou Óptico, 28046 Madrid, Spain; a.glezabad@hotmail.com (A.G.-A.); adiaz@afflelou.es (G.I.A.-F.)

**Keywords:** myopia, childhood, prevalence

## Abstract

Background: In recent years, there was a significant increase in myopia incidence worldwide. However, it is still not clear how it affects Spanish children. Since 2016, this research team analyzed myopia prevalence and risk in 9668 children aged between 5 and 7 years. It was shown that the prevalence rates increased from 16.8% in 2016 to 20.4% in 2019. The objective of this study is to update the prevalence rate of myopia in Spain in 2020 and analyze the risk and prevention factors of myopia. Methods: The participants underwent an optometric examination, and a questionnaire on their lifestyle, family history, and geographical origin was carried out. Finally, data were analyzed using the SPSS version 27 program. Results: 1601 children from various Autonomous Communities of Spain were examined. In 2020 the myopia rates did not increase compared to 2019 (*p* < 0.05), although the number of hyperopes decreased and the number of emmetropes increased. Regarding age, the prevalence of myopia increased progressively over the years (*p* < 0.001). There was no association between gender and myopia (*p* > 0.05). There was a link between the time spent in near vision and family history with the prevalence of myopia (*p* < 0.05). Conclusions: The prevalence of myopia in Spain in children between 5 and 7 years old increased significantly between 2016 and 2020.

## 1. Introduction

Myopia is the most common refractive error worldwide. It is estimated that 22.9% of the world population is myopic, of whom 2.7% have high myopia [1,2,3,4]. Various investigations have found that most cases of myopia develop during school age, with the prevalence increasing after age 6 years [5,6,7]. These numbers are expected to rise in the coming years; thus, in 2050, 49.8% of the world population is expected to be myopic and 9.8% will have high myopia. In addition, in the age range between 5 and 9 years, it is estimated that the myopia rate will double, from 5% in 2000 to 10% in 2050 [2].

When analysing the impact of myopia on the quality of life, it was seen that visual impairment is associated with a significant reduction in activities of daily life, intense visual tasks, and a decreased level of participation in society. This can harm education, employment, child development, mental health, and functional ability in older people. If myopia is not corrected, it can affect children’s academic performance [8]. A recent estimate suggests that visual impairment among preschool children will increase by 26% by 2060 and that uncorrected refractive error will account for 69% of cases [9].

Various studies have indicated that the prevalence of myopia in children varies according to ethnicity age and geographical origin. According to the WHO (World Health Organization), the percentage of myopia in subjects younger than 20 years old in Southeast Asia is 4.9% (1.6–8.1), in the West Pacific it is 18.2% (10.9–25.5), in Africa 6.2% (4.8–7.6), in America 8.4% (4.9–12), and 9.2% in the Eastern Mediterranean (10.5–12) [10]. In Europe, the number of studies on the prevalence of myopia in children is very limited. In the Aston Eye Study (AES), the percentage of myopic persons in England at the age of 6–7 years old is 9.4% versus 29.4% at the age of 12–14 [11]. In turn, ethnic differences have been found, with a higher prevalence in children of Asian origin (36.8%) compared to white European children (18.6%). In Ireland, in subjects of the same age, the myopia rate increased from 2.8% to 17.7% between 2010 and 2015 [12]. In the Netherlands, the prevalence is lower, with only 2.4% of children being myopic at 6 years old [13]. In Poland, the percentage increases from 2% at 6 years old to 8.4% at 8 years old and to 14.7% at 12 years old [14].

Although it was shown that there is an upward trend in the prevalence of myopia in children worldwide, it is still not clear how it affects Spanish children. For this reason, since 2016, this research team has analysed the prevalence of myopia and the risk factors in children aged between 5 and 7 years old. In the study published in 2019, in which 6152 children participated, it was found that the prevalence of myopia increased from 16.8% in 2016 to 19.1% in 2017. In turn, it has also been found that lifestyle seems to increase the risk of myopia [15]. Taking into account that between 15th March and 21st June 2020 in Spain there was a period of confinement due to the Coronavirus Disease 2019(COVID-19) pandemic, this study offers an update of the myopia rate and its relationship with lifestyle.

## 2. Materials and Methods

### 2.1. Study Design

This is an epidemiological study with a cross-sectional design. Data collection was carried out in Alain Afflelou optical centres in the different autonomous communities of Spain.

### 2.2. Study Population

The study population consists of children between 5 and 7 years old who participated in the “School campaign in favour of children’s visual health”. This campaign is aimed at all schools in Spain, so all children between 5 and 7 years old who are starting their school stage and who were interested in participating were included in the study.

In turn, the parents of all the children who participated in this research had to read the information sheet and sign the informed consent form before the data collection began.

The selection of the participants was carried out by means of convenience sampling and non-probability sampling. In other words, the recruitment of participants was on a voluntary basis and all of the children who participated in the campaign were included. In this way, it was possible to obtain information on the refractive state and the lifestyle of children between 5 and 7 years old in a fast and accessible manner.

### 2.3. Inclusion and Exclusion Criteria

All of the children who participated in the study had to be between the ages of 5 and 7 years old. In addition, the parents were required to understand and sign the informed consent form.

With regards to the exclusion criteria, the following cases were excluded: poor collaboration on the part of the patient, the age of the participant was not within the established range, the optometric review was carried out outside the campaign, and the files were incomplete and/or the questionnaire had not been completed.

### 2.4. Clinical Procedure

All of the children underwent an optometric examination, which consisted of a questionnaire and an evaluation of refraction and binocular conditions:

Questionnaire: this was divided into six sections, which included questions about personal data (city of residence, age, sex, and nationality), family ocular health history, lifestyle (extracurricular activities and number of hours/weeks dedicated to carrying out these activities, time dedicated to the use of devices and genetics), and anamnesis (symptoms, main complaint, diagnosis or ocular previous treatment, medication and systemic diseases, and date of the last revision).

Optometric examination: The standard procedure was as follows:

(1) Best corrected and uncorrected visual acuity.
(2) Objective refraction: non-cycloplegic retinoscopy (differences of ±0.5D were estimated in the spherical equivalent compared to the non-cycloplegic refraction) [16].
(3) Subjective refraction.
(4) Binocular vision and accommodation tests: Cover–uncover alternating cover, ocular motility, Hirschberg test, Worth test, near point of convergence, amplitude of accommodation, stereopsis, and colour vision.
(5) Finally, the anterior segment (eyelid, eyelashes, lid margin, cornea, conjunctiva, and lens) was reviewed using a slit lamp.

### 2.5. Definition of Variables

To determine the refractive error in children, the criterion of the spherical equivalent (SE) was used. The formula was SE = sphere + cylinder/2. Refractive errors have been defined as follows: hyperopia: SE > +0.5D; myopia: SE ≤ −0.5D; emmetropia: −0.5D < SE < +0.5D. Within myopia, a subdivision of the degree of myopia was made, according to the classification of the American Academy of Optometry [17]: low myopia: −0.5D > SE > −3D; moderate myopia: −3D > SE > −6D; high myopia: SE ≤ −6D.

To determine the number of hours per day spent exposed to sunlight, according to the classification made by the Clinical Myopia Profile [18], three ordinal categorical variables were defined: low (between 0 and 1.6 h/day), moderate (between 1.6 and 2.7 h/day), and high (>2.7 h/day).

With regards to the number of hours per day in near vision, the same classification was used, for which three ordinal categorical variables were defined: low (between 0 and 2 h/day), moderate (between 2 and 3 h/day), and high (>3 h/day). Within this time interval, the percentage of use of digital devices was established using three ordinal categorical variables: <25%, between 25% and 50%, and >50%.

### 2.6. Statistical Analysis

The statistical analysis was carried out using SPSS 27.0 software (SPSS Inc., Chicago, IL, USA). The normal distribution of the variables was verified with the Kolmogorov–Smirnov test, with a significance level equal to 0.05. Since the null hypothesis of the normal distribution was rejected based on the Kolmogorov–Smirnov test, nonparametric statistics were applied in order to evaluate the statistical significance in the variable “refractive error” in the different groups and to determine the significant relationship between risk factors with respect to myopia. The prevalence was calculated with a 95% confidence interval. In turn, to assess statistical significance, a cut-off point of *p* ≤ 0.05 was considered.

## 3. Results

### 3.1. Demography

In 2020, a total of 1635 children from all the autonomous communities of Spain participated, of which 32 medical records were excluded because they did not meet the inclusion criteria or the records were incomplete. The total number of retained samples was 1601. The mean age was 6.13 ± 0.79 years.

**Figure 1:**
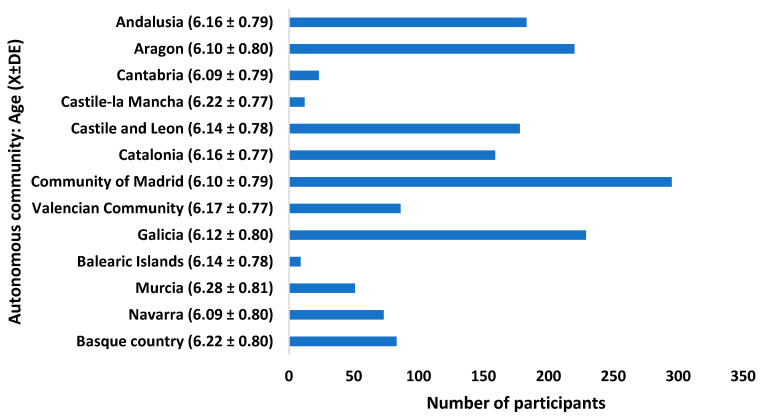
Frequency of participants by CA.

With regard to sex, 49.5% were boys and 50.5% were girls. Figure 1 shows the number of participants and the average age in each Autonomous Community.

### 3.2. Prevalence Results

**Figure 2:**
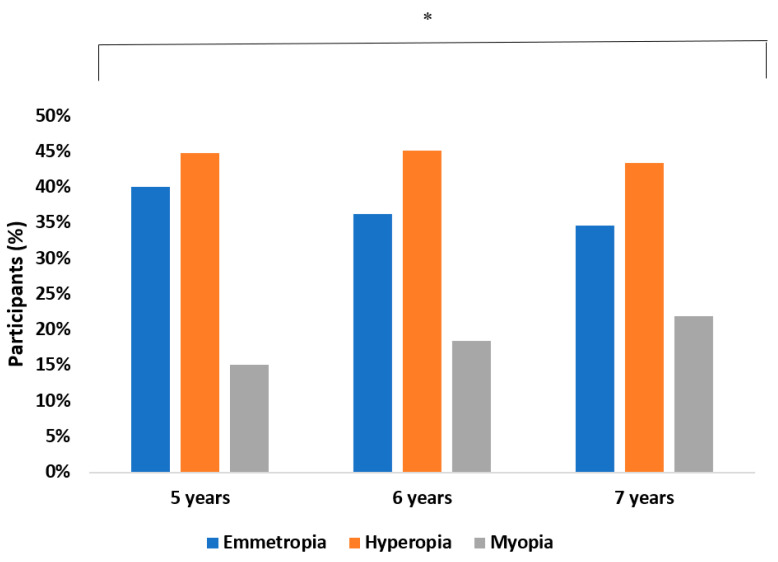
Prevalence of refractive errors as a function of age. * p < 0.005.

In 2020, the prevalence of myopia was 20.1%. It was found that between 2019 and 2020, the myopia rate did not change (*p* > 0.05; CI: 0.80–0.83). With regards to sex, 21.2% of boys and 17.6% of girls were myopic. No statistically significant differences were found between gender and the risk of having myopia (OR: 0.46; CI: 0.40–0.51; *p* > 0.05). The myopia rate increased significantly with age (*p* ≤ 0.001) (Figure 2).

Regarding the autonomous communities, no statistically significant association was found between the risk of having myopia and the geographical dwelling location (OR: 7.14; CI: 6.73–7.55; *p* > 0.05).

#### 3.2.1. Distribution of the Mean Value of the Spherical Equivalent

The mean value of the spherical equivalent for the total number of participants was 0.48 ± 1.77D. Regarding sex, the mean and SE were 0.43 ± 1.83D in boys and in girls, 0.54 ± 1.72D. In relation to age, there was a clear tendency toward myopia with age (*p* ≤ 0.001). Thus, at age 5 years old, the mean and SE were 0.56 ± 1.91D, at 6 years old, 0.56 ± 1.69D, and at 7 years old, 0.37 ± 1.76D.

#### 3.2.2. Degree of Myopia

Of all participants with myopia, 88.8% (*n* = 277) had low myopia (−0.5D > SE > −3D), 9.0% (*n* = 28) had moderate myopia (−3D > SE > −6D), and 2.2% (*n* = 7) had high myopia (SE ≤ −6D). As shown in Table 1, no significant association was found between the degree of myopia with respect to gender and age (*p* > 0.05).

### 3.3. Risk Factor Results

#### 3.3.1. Near-Vision Activities and the Use of Digital Devices

Of the total number of participants, the time spent in near vision according to the classification used was as follows: in 16.5%, low (between 0 and 2 h per day), in 41.7%, moderate (between 2 and 3 h/day), and in 41.9%, high (>3 h/day). In 2020, the children who spent a moderate amount of time in near vision increased significantly (*p* ≤ 0.001; CI: 0.74–0.80).

Within the time interval in near vision, 17.4% of the participants spent less than 25% of the time using digital devices, 40.7% between 25–50%, and 41.8% spent more than 50% of their time. In 2020, a significant increase was seen in the use of digital devices compared to other years (OR: 1.26; CI: 1.23–1.29; *p* ≤ 0.001).

**Figure 3:**
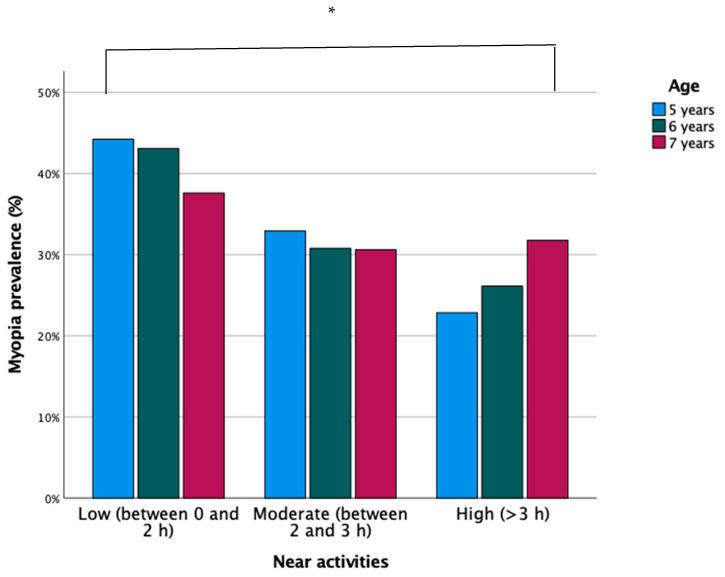
Prevalence of myopia according to time in near vision and age. * p < 0.005.

**Figure 4:**
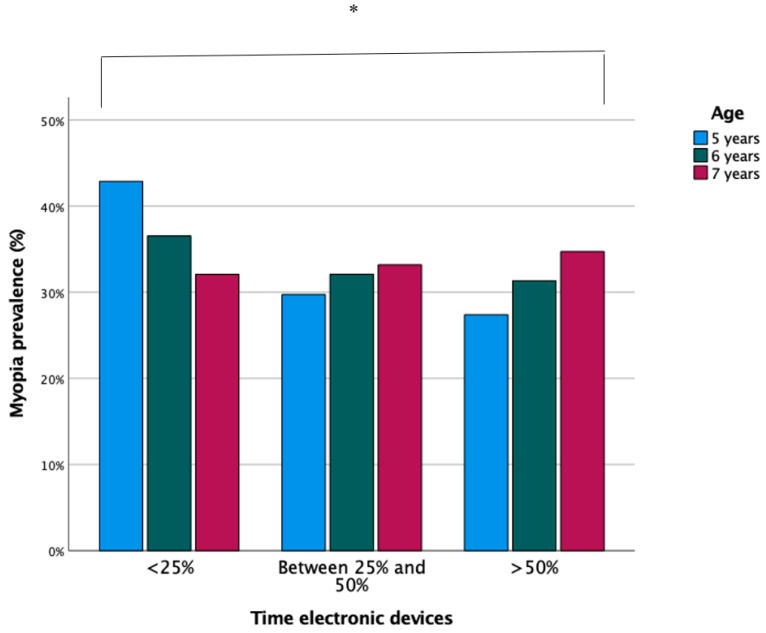
Prevalence of myopia according to the use of digital devices and age. * p < 0.005.

The time spent in near vision and the use of devices increased with age (*p* ≤ 0.001). Therefore, as shown in Figure 3 and Figure 4, there was an association between the time spent in near vision and the use of digital devices with the increase in children with myopia (OR: 0.87; CI: 0.85–0.90; *p* ≤ 0.001).

#### 3.3.2. Family Background

Of the total number of participants with myopia, in 35.1% of the cases, neither parent was myopic, in 18.1% of the cases, both parents were, and in 46.7% of the cases, one of the parents was myopic. Therefore, a relationship was found between the presence of myopia in one or both parents and the refractive state of the children (*p* ≤ 0.001).

A myopic trend was found in the mean and SE values according to the presence of myopia in the parents. That is, the risk of having myopia increased depending on whether one or both parents were myopic (OR: 1.28; CI: 1.15–1.41; *p* ≤ 0.001).

### 3.4. Protection Factor Results

**Figure 5:**
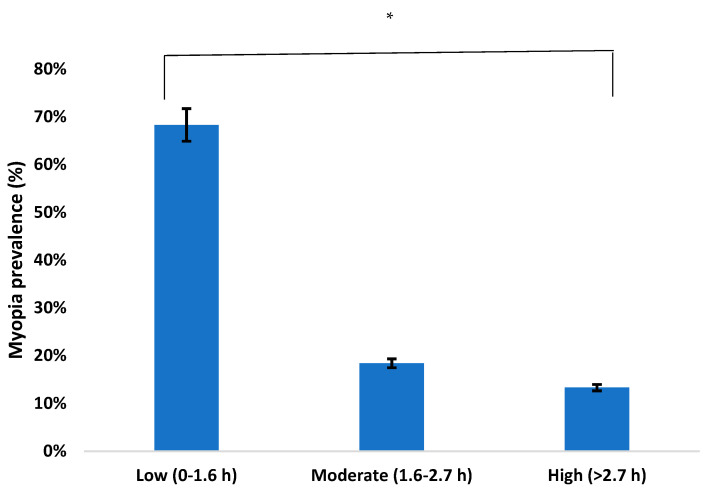
Prevalence of myopia according to time spent outdoors. * p < 0.005.

The amount of time children spent outdoors each day was analysed, that is, the number of hours per day they were exposed to sunlight. Of the total participants, 68.3% spent between 0–1.6 h/day, 18.4% between 1.6–2.7 h/day, and 13.3% more than 2.7 h/day. As shown in Figure 5, the prevalence of myopia decreased the longer the time of exposure to sunlight (*p* ≤ 0.001).

## 4. Discussion

The prevalence of myopia in children between 5 and 7 years old in Spain has increased significantly in recent years, from 16.8% in 2016 to 20.1% in 2020. If we compare these results with other studies carried out in Spain in previous years, it can be seen that from 2000 to 2020 the prevalence of myopia increased by 17.6% (2.5% in 2000 and 20.1% in 2020) [19]. In the study by Antón et al. [20], the incidence was 25.4% between age 49 and 79 years in 2009, a number which is very similar to that in 2019 and 2020 in children aged 5 and 7 years old. This corroborates the significant increase in myopia in Spain in recent years, since the incidence in adults in 2009 is the same as that in children in 2020. It should be noted that between 2019 and 2020, the prevalence of myopia did not increase. However, in the last year with respect to the previous ones, there was a trend towards emmetropia and a decrease in hyperopia. As in other studies, it must be considered that the greatest increase in myopia occurred between 2016 and 2017; since then the trend has decreased significantly [21,22,23]. Depending on the geographic location, the prevalence of myopia in children and adolescents is different. Thus, in Africa, the myopia rate is lower than our results, 3.2% in subjects age between 12 and 15 years old [24]. In Brazil, the myopia rate is 2.8% at 6 years old and 4% at 10 years old [25], and in Colombia, 11.2% between 8 and 17 years old [26]. On the contrary, in some Asian countries, myopia rates are higher, reaching peaks of 80–90% in some populations [27]. Németh et al. [28] found that in the last six years, the prevention of myopia in Asia increased by 90% compared to 55% in European countries. This difference in myopia incidence rates may be due to a country’s economic position and lifestyle. If the more developed Asian countries are taken as a reference, a high prevalence of myopia in children can be observed. In Spain, the way of life of children is increasingly similar to that of Asia, so myopia rates in a few years may increase to reach these numbers. Spain is one of the European countries with the highest level of concern about the increase in myopia. In recent years, Spain, together Russia and Portugal, has increased the perceived efficacy of the various myopia control methods, mainly soft lenses for myopia control and orthokeratology and therefore, an increase in adaptations [29]. This is related to a reduction in the rate of myopia in children during the last year. However, when comparing the prevalence of myopia in different countries, the value of the spherical equivalent (SE) used must be considered. Thus, in the study carried out in California, considering a more negative SE value (SE ≤ 1.0D), the myopia rate may be underestimated with respect to our cut-off point (SE ≤ −0.5D).

The influence of gender on the prevalence of myopia was analysed in numerous studies. However, the results vary since depending on the country, the incidence rate was higher in males or females or was the same in both sexes. When analysing different years, statistically significant differences between the prevalence of myopia and gender were not found. Despite the fact that in 2016 the percentage of myopic boys was slightly higher, although not significant, with respect to girls, these number have become similar over the years. However, other studies found a higher prevalence in girls or boys, associating it with their school performance [30,31,32,33]. This may be due to the fact that the relationship between myopia and sex is different depending on the society in which a child lives, so it can be influenced by both the environmental and biological factors. In addition, in the past, girls spent a lot of time indoors and generally did less sports and outdoor activities compared to boys, which is why myopia rates were higher in this gender. Nowadays, as was obtained in our results, both sexes carry out the same activities equally; for this reason, no gender differences have been found in Spanish children.

With regard to lifestyles, this study revelaed that the longer the time spent in near vision and using digital devices, the higher the prevalence of myopia, and this time increases progressively with age. Thus, between 2016 and 2017, most of the children spent >50% of the time in near vision, percentages that decreased significantly between 2019 and 2020. This is related to a stabilisation of the myopia rate. Similar results have been found in the studies by Lin et al. [34] and Theophanous et al. [22]. Thus, the use of computers, reading time, and working distance are associated with the development of myopia [35]. At the same time, other research has associated an increased risk of developing myopia with reading at short distances (<20 cm) and for continuous periods of time (>45 min), instead of associating them with the total time spent on all activities [36,37]. However, other investigations have not found a relationship between near work and myopia [38,39]. In the study by Lin et al. [34], who examined 386 children between 6 and 17 years old, the results did not find a higher rate of myopia in both children and adolescents who spent a long period of time in near proximity. Currently, in Spain, children spend many hours each day doing activities in near proximity both at home and at school. This implies that outdoor activities are few, so programs should be established in schools to improve children’s lifestyles and thus reduce the increase in the prevalence of myopia. When comparing our results with the data in the existing bibliography, it can be said that near-vision work and the excessive use of digital devices are a risk factor for the appearance and development of myopia. However, there is still not enough scientific evidence to determine the cause, namely, the time spent in near vision or working distance. In addition, this study found that since 2017, the number of hours spent using digital devices has decreased. This may be due to a better awareness of the risk on the part of the parents. In turn, this leads us to think that in the coming years the prevalence of myopia may be reduced by better exposure to the open air and a shorter time in near vision. However, it should be noted that the significant increase in the number of hours in close proximity and with digital devices between 2019 and 2020 is due to the fact that during last year, children spent more time at home due to the COVID-19 pandemic.

Many epidemiological studies have shown a significant association between the prevalence of myopia in parents and the risk of a child developing myopia [40]. In our results, a relationship was found between the presence of myopia in one or both parents and the refractive state of the children. Similar results were found in the study by Jiang et al. [41]: according to the number of myopic parents, the risk of the child having myopia can be predicted.

Time outdoors is considered a protective factor that reduces the development of myopia. In our study, it was found that the prevalence of myopia decreases the longer the exposure to sunlight. Similar results were found by Deng et al. [42]. Furthermore, Lingham et al. [43] also found that spending more time outdoors in childhood is associated with a reduced risk of myopia in young adulthood. That is, spending more time outdoors in both childhood and adolescence is associated with less myopia in young adulthood. As expected, in this study it was found that the time of outdoor exposure increased from 2016 to 2020. This increase may be due to the fact that in recent years, as shown in the study by Zhou et al. [44], an attempt was made to make parents aware of the importance of children spending more time outdoors. Thus, the improvement of children’s attitudes is thanks to the help of parents and favours a reduction in myopia during school age; 2019 was the year when the greatest increase occurred. It should be noted that during 2020 due to the COVID-19 pandemic, in Spain there was a confinement period of four months. Therefore, during this year, the time of outdoor activities was reduced. There is a difference between the autonomous community where children live and the time spent outdoors. Thus, in Galicia, Valencia, and Cantabria, children spend more than three hours each day exposed to sunlight. This may be due to the fact that in the most industrialised communities, the time spent in near vision is much higher and therefore children do few activities outdoors. In 2020, in the Basque Country, 40.2% of children spent >2.7 h/day outdoors, a higher number than in the rest of the communities across different years.

With regard to the limitations of our study, it can be highlighted that in the 2018 campaign, the number of participants was much lower compared to the other years. The low participation of the centres of Asturias, the Canary Islands, and Ceuta excluded them from the study. However, when analysing the different years, the existing progression of the prevalence of myopia in Spain was seen. In addition, the refraction that was carried out was without cycloplegia because in Spain the use of cycloplegic drugs by optometrists is forbidden. However, the results represent a very large sample size to be able to estimate the prevalence of myopia. Furthermore, the selection of the sample was not carried out randomly, but through convenience sampling, and the campaign offered free glasses for children who needed them, which could lead to a bias in the results.

## 5. Conclusions

The prevalence of myopia in Spain in children between 5 and 7 years old increased significantly between 2016 and 2020. In addition, a tendency to myopisation was found the longer the time in near vision and with the use of electronic devices. The prevalence of myopia decreases with longer exposure to sunlight.

## Figures and Tables

**Table 1:** Frequency and percentage of degree of myopia according to age and gender.

Gender	Age	Degree of Myopia		
		Low *n* (%)	Moderate *n* (%)	High *n* (%)
Female	5	21 (16.2)	4 (33.3)	0 (0.0)
	6	49 (37.7)	4 (33.3)	1 (50.0)
	7	60 (46.2)	4 (33.3)	1 (50.0)
	Total	130 (100.0)	12 (100.0)	2 (100.0)
Masculine	5	25 (17.1)	4 (25.0)	2 (40.0)
	6	60 (41.1)	2 (12.5)	0 (0.0)
	7	61 (41.8)	10 (62.5)	3 (60.0)
	Total	146 (100.0)	16 (100.0)	5 (100.0)
Total	5	46 (16.7)	8 (28.6)	2 (28.6)
	6	109 (39.5)	6 (21.4)	1 (14.3)
	7	121 (43.8)	14 (50.0)	4 (57.1)
	Total	276 (100.0)	28 (100.0)	7 (100.0)

## Data Availability

The data presented in this study are available on request from the Alain Afflelou Foundation.
